# Supplementary material for: Study on the Effect of EZH2 Inhibitor Combined with TIGIT Monoclonal Antibody against Multiple Myeloma Cells
Source: Int J Mol Sci. 2023 May 11;24(10):8603. doi: 10.3390/ijms24108603 (PMC10218613; doi:10.3390/ijms24108603)
Supplement: Supplementary file 1 [file ijms-24-08603-s001.zip › Supplementary Figures.pdf]

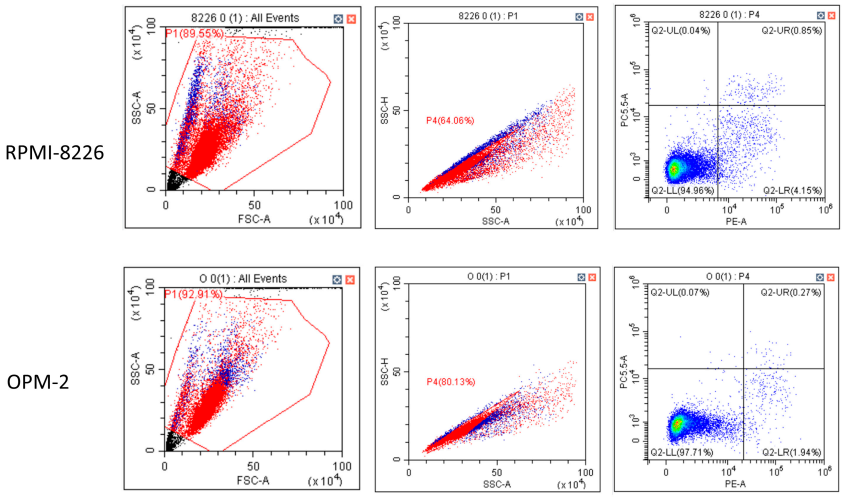

Figure S1. Gate strategy for setecting apoptosis of OPM2 and RPMI-8226 cells by flow cytometry.

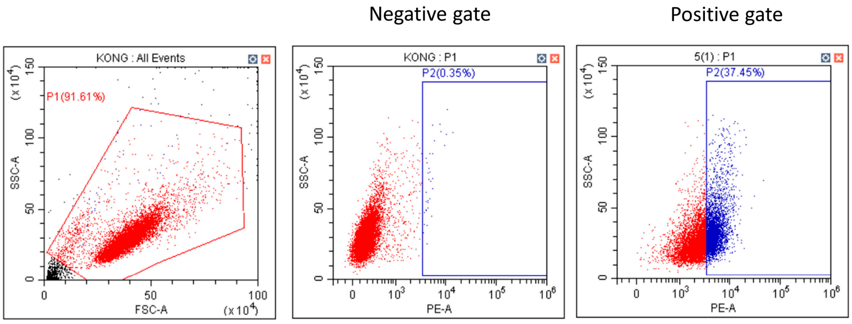

Figure S2. Gate strategy for detecting CD155, CD112 and CD113 expression on OPM2 cells by flow cytometry.

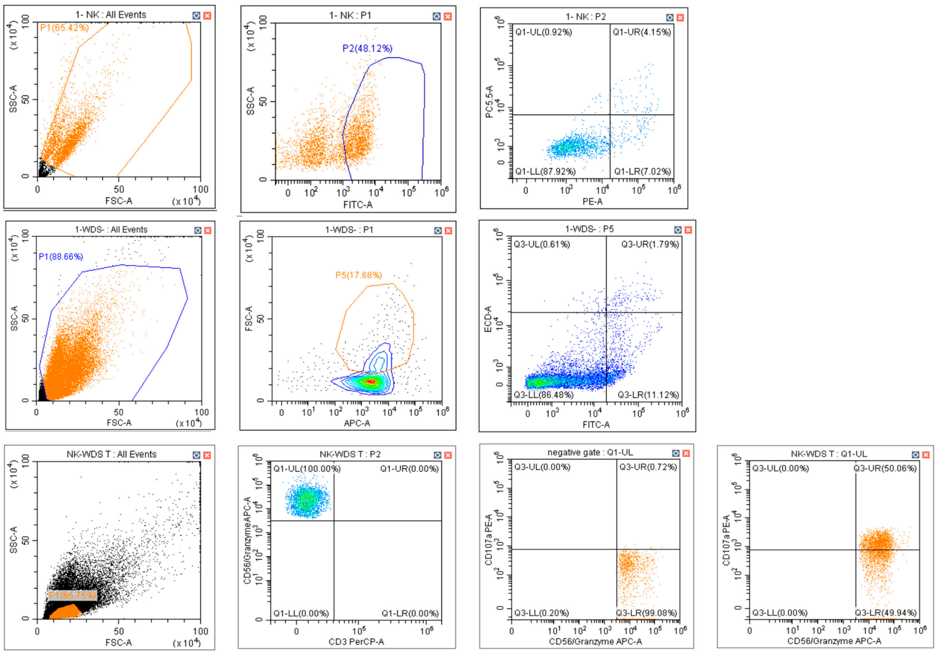

Figure S3. Gate strategy for detecting targeting cells apoptosis and NKP30 expression on NK cell surface in co-culture system by Flow cytometr.
